# Attitude of Health Care Workers (HCWs) toward Patients Affected by HIV/AIDS and Drug Users: A Cross-Sectional Study

**DOI:** 10.3390/ijerph14030284

**Published:** 2017-03-09

**Authors:** Caterina Ledda, Francesca Cicciù, Beatrice Puglisi, Tiziana Ramaci, Giuseppe Nunnari, Venerando Rapisarda

**Affiliations:** 1 Occupational Medicine, Department of Clinical and Experimental Medicine, University of Catania, Via Santa Sofia, 95123 Catania, Italy; vrapisarda@unict.it; 2 University Hospital of Catania Policlinico-Vittorio Emanuele, Via Santa Sofia, 95123 Catania, Italy; francescacicciu@gmail.com (F.C.); beatricepuglisi@hotmail.it (B.P.); 3 Faculty of Human and Social Sciences, Kore University of Enna, Viale delle Olimpiadi, 94100 Enna, Italy; tiziana.ramaci@unikore.it; 4 Unit of Infectious Diseases, Department of Clinical and Experimental Medicine, University of Messina, Via Consolare Valeria, 98124 Messina, Italy; giuseppe.nunnari@unime.it

**Keywords:** healthcare workers, HIV, AIDS, drug users

## Abstract

Caring for HIV/AIDS patients and/or drug users requires health care workers (HCWs) to have good knowledge of the issues. Cultural differences in HCWs, combined with professional ethics and personal beliefs, could also result in conflicting attitudes, leading to difficulties related to looking after people affected by HIV/AIDS or drug users. A cross-sectional study was carried out to assess the attitude towards HIV/AIDS patients and/or drug users in a sample of workers operating in a large university hospital in southern Italy. A total of 736 workers were surveyed from May to November 2016. During the periodic occupational health surveillance, a questionnaire was administered about attitudes of discrimination, acceptance and fear towards these patients. Respondents showed average levels of acceptance to HIV/AIDS and drug user patients. As years of experience and professional training increased, scores for discrimination, acceptance of HIV/AIDS, acceptance of drug users and fear decreased. Factors positively influencing levels of attitudes were being female and younger. Supplementary education is needed to strengthen the awareness of HCWs.

## 1. Introduction

People living with HIV/AIDS require ongoing health care services as they are potentially at increased risk of developing disorders including cardiovascular and liver disease, accelerated bone loss, metabolic disorders, etc. [1,2,3]. Those able to access medical care are living longer and have improved their health thanks to antiretroviral medications. These patients are experiencing acute HIV-related chronic episodes and other types of illness that can require hospitalization and/or supportive care arrangements [4,5]. Several studies have investigated the attitudes, knowledge and practices of health care workers (HCWs) towards patients with HIV/AIDS and underlined that HCWs still fear the disease and behave prejudicially toward HIV/AIDS patients [6,7,8,9,10,11].

Factors which influence these attitudes include fear of contagion associated with the uncertainty of care and the awareness of feeling useless in providing care for patients with a potentially fatal disease [12].

Drug users are hospitalized more frequently than the general population [13,14,15,16]. Within hospitals, people who use substances encounter significant barriers to access care [4,17]. They are often labeled as being “challenging, manipulative, drug-seeking and demanding” by HCWs [18,19]; they also meet difficulties, receive substandard care and frequently leave hospitals against medical advice [20,21,22].

Taking care of HIV/AIDS patients and/or drug users requires HCWs to have good knowledge of their unique issues. Cultural differences in HCWs, combined with professional ethics and personal beliefs, could also result in conflicting attitudes, which may lead to difficulties related to caring for HIV/AIDS patients and/or drug users [4,12].

The purpose of this study was to assess the attitude towards patients affected by HIV/AIDS and/or drug users in a sample of HCWs operating in a large university hospital in southern Italy.

## 2. Materials and Methods

A cross-sectional study was conducted from May to November 2016 at the University Hospital of Catania (Italy) where the health care workforce consists of ≅2800 HCWs.

The study was performed as part of the periodic occupational health surveillance and it required no formal approval by the local ethics committee, which was nevertheless consulted and granted its informal authorization. Participants were informed about the study aims and procedures and gave their written informed consent to participate.

This study was conducted using a self-administered multiple-choice questionnaire developed by See et al. [23]. This tool allows us to evaluate four aspects: discrimination, acceptance of HIV/AIDS patients, acceptance of drug users and fear. For each of the four aspects there were four multiple choice questions with answers ranging from: “strongly disagree” = 0, “disagree” = 1, “agree” = 2, “strongly agree” = 3. Negative questions were reverse-scored to ensure that the direction was consistent with all items and higher scores represented a more positive professional attitude [23]. The original version of the questionnaire was translated into Italian by an expert mother tongue translator.

A further section was added in order to collect information about age, gender, schooling, professional training and occupational history.

After emphasizing the importance of the topic, the questionnaire was explained and distributed by two trained occupational physicians, then the self-administered questionnaires were completed by the HCWs in an anonymous and voluntary manner.

In order to proceed with a statistical analysis, HCWs were grouped according to their tasks: physicians, graduate sanitary (biologists, physicists, chemists, psychologists); nurses and midwives; healthcare assistance staff (physiotherapists, orthoptists, logotherapists); healthcare diagnostic staff (lab technicians, radio technicians, audiometric technicians, neurophysiopathology technicians). Then, employees were divided by work environment: surgery, medicine and services. The latter include support departments like anesthesiology, radiology, laboratories.

Data were analyzed with the software SPSS 22.0 (SPSS Inc., Chicago, IL, USA) for Windows. Descriptive analyses were performed using frequencies’ percentages. Scores were reported as mean ± standard deviation (SD).

For the bivariate analysis, *t*-tests were performed to evaluate differences in quantitative variables. The one-way variance analysis (ANOVA) was used to determine any statistically significant differences between the groups’ means.

A logistic regression model was used to detect possible factors associated with attitude status and age, gender, professional training and service years. Moreover, gender and age, as possible confounders, were included in the regression model when analyzing professional training and service years. Results are expressed as odds ratio (OR) with 95% CI. The level of significance was set at *p* ≤ 0.05.

## 3. Results

In this study, 736 HCWs were examined by university occupational doctors; all participants were asked to complete the questionnaire, which was administered to 713 workers (97% response rate), whereas 3% (*n* = 23) of the HCWs refused to participate in the survey, owing to lack of time needed to reply to questions.

A total of 353 (48%) were male, with a mean age of 41.2 ± 16.7 years and a duration of employment of 15.4 ± 12.3. The characteristics of the sample are reported in Table 1. 

Over 80% of the sample was made up of physicians (40%) and nurses and midwives (42%). The remaining part (18%) was health care assistance staff, health care diagnostic staff and graduate sanitary. However, the sample was equally distributed in a cross three work environments: surgery, medicine and services.

Table 2 shows the scores of the questionnaires. Female HCWs showed more statistically significant acceptance towards drug users than male HCWs, and expressed no negative emotions such as discrimination and fear.

As age increased, a progressive reduction of the scores was observed in all four aspects: discrimination, acceptance of HIV/AIDS patients, acceptance of drug users and fear.

The ANOVA test showed statistically significant reductions in age bands >30 years as to discrimination and acceptance of HIV/AIDS, and >40 years as to the acceptance of drug users and ≥50 as to fear.

There is a statistically significant gap between physicians/nurses/midwives and the remaining investigated staff in all four aspects. The former show significantly greater discrimination and fear, with significantly less acceptance and fear, and so do those working in the surgery department and services areas.

The health care assistance staff only showed a significant score in relation to fear.

The results of the logistic regression (see Table 3) pinpoint how male subjects show greater discrimination and fear than female workers. Being over 40 is considered a risk factor related to a discriminatory attitude, acceptance of drug users and fear in general. After 50 years old, the workers’ level of acceptance of HIV/AIDS patients decreases. 

Having more than three years of professional training in biological risks and drug user management turns out to be a risk factor, given all the attitudes analyzed. The same happens for those with more than 11 years of service.

## 4. Discussion

The questionnaire used in this survey was developed by See et al. [23] and modified for the purpose of this study. The response rate was 97%, and thus it well represents the sample analyzed.

As observed by See et al. [23], the questionnaire was reliable and valid for assessing the professional attitude of HCWs towards serving HIV/AIDS patients and/or drug users.

The results allow us to observe that discrimination and fear scores significantly decreased among HCWs by age, suggesting poorer professional attitudes within these domains, while the acceptance of HIV/AIDS and/or drug users decreased if the sample age increased.

From the data of our study it is observed that being older than 40 seems to be a significant risk factor in terms of discriminatory attitudes, low tolerance towards drug users and the generation of fear.

Furthermore, starting from 50 years of age, employees manifest poor tolerance (acceptance) of HIV/AIDS patients.

Similar results have been reported by other surveys [6,7,8,9,10,11] that dealt with HCWs’ attitudes towards HIV patients.

Logistic regression highlighted how male workers show discrimination and fear attitudes to a greater extent than female ones. These data were observed for the first time after this survey.

The analysis of the scores to the 16 questions, asked in relation to the role/task of each HCW, allows to detect that physicians, nurses and midwives show similar values. This, as already reported in a previous survey conducted by See et al. [23], showed that HCWs, who are more exposed to biological risks, accept HIV/AIDS patients and drug users in a similar manner.

Although HCWs had negative feelings about HIV/AIDS patients [24], their integrity allowed them to overcome their fear of HIV infection [25,26]. Thus, HCWs accept both HIV/AIDS patients and drug users in a similar way [23].

In the same fashion, the years of service seem to be an unfavorable factor, as HCWs with more than 11 years of service showed greater discriminatory attitudes, poor tolerance towards HIV/AIDS patients and drug users and more fear. These data are in line with the observation according to which older workers develop significantly negative feelings towards these kinds of patients compared to younger HCWs.

It may be that such results may be accounted for by the fact that the cultural environment they grew up in allowed younger HCWs to get to know these pathologies more deeply, as well as the social levels where it is usually possible to detect them [7].

Indeed, many people believe that HIV/AIDS is contracted due to immoral behavior, such as risky sexual intercourse and illegal use of drugs [20,23].

Predictors for a positive attitude were previous experience in caring for HIV/AIDS patients, age and having no children [10].

Besides, as far as the information/education of HCWs about biological risks and drug user management is concerned, they seem to have an essential role, since there are better results in those employees who received specific training less than three years before. Additionally, the scores regarding discrimination, tolerance and fear were significantly lower.

In a recent survey on nurses conducted by Marranzano et al. [8], it was observed that training, competence in the care of patients with HIV/AIDS and the prevention of HIV-related occupational risks are of paramount importance.

In the same way, several studies have shown that the competence of physicians and nurses in the care and prevention of alcohol and drug addiction is strategically important [27,28,29].

Unlike many other diseases, HIV/AIDS cannot be cured. Its symptoms can become severe, and it is associated with stigma. Hence, many HCWs are afraid of contact with HIV/AIDS patients [9,10]. In the first 10 years after HIV/AIDS appeared, many studies discussed the reluctance of HCWs to have contact with HIV/AIDS patients due to fear of contracting the virus [10,11,12].

As far as drug and alcohol addicts are concerned, many physicians and nurses took on a reluctant behavior in approaching these patients, fearing they could lie about their use of substances [27,30,31]. The score of fear in this study indicates that HCWs are still scared of HIV patients’ and drug users’ behavior.

As observed in other studies, the positive attitudes of HCWs have been correlated with high levels of HIV/AIDS and alcohol consumption knowledge [5,10,11,12,13,19], and also through on-going training [8].

Moreover, as described by others, it is important to examine not only the effectiveness of the program in changing the awareness, attitudes and behavior of HCWs, but the durability of its impact, even through follow-up program carried out every six to 12 months [8,10,25].

Drawbacks of the present study relate to getting results from a single hospital; therefore, they might not reflect HCWs’ attitudes and concern in other hospitals in Italy, even though the results are comparable to those observed in other surveys [6,7,8,9,10,11,23].

Furthermore, despite the questionnaire being self-administered, the intervention of two occupational physicians may have partially influenced the participants’ responses.

## 5. Conclusions

In conclusion, in order to improve HCWs’ attitude towards patients, there are several possibilities that involve various professional figures, i.e., toxicologists, infectivologists, psychologists, etc. As far as the occupational medicine competence area is concerned, it seems to be of great importance to set up intense education programs, to spread information about contagion pathways, and to use general and personal protection devices by adopting safety procedures in accordance with internationally recognized organizations’ guidelines, preventing accidents and/or inconveniences to patients.

In order for these education programs to effectively reach the HCWs, they should be put among the hospital’s strategic objectives by the hospital management. Such programs should be mandatory every three years and their attendance should confer credits to the participants’ skills and acquired job-content knowledge and implementation; besides, follow-ups should be carried out every six months.

## Figures and Tables

**Table 1:** Sample characteristics.

Variables	Results
**Gender**	
Male	353 (48%)
**Age**	
≤30	136 (18%)
30–39	189 (26%)
40–49	213 (29%)
≥50	198 (27%)
**Health care workers (HCWs) subgroup**	
Physician	293 (40%)
Graduate sanitary	23 (3%)
Nurse and midwife	309 (42%)
Healthcare assistance staff	60 (8%)
Healthcare diagnostic staff	51 (7%)
**Schooling**	
Bachelor’s Degree	363 (49%)
Master’s Degree	57 (8%)
Post-graduate specialization	234 (32%)
PhD	82 (11%)
**Work environment**	
Surgery	262 (35%)
Medicine	248 (34%)
Services	226 (31%)

**Table 2:** Scores obtained from HCWs.

Variables	Discrimination Scores (0–4)	Acceptance of HIV/AIDS Scores (0–4)	Acceptance of Drug Users Scores (0–4)	Fear Scores (0–4)
**Gender**				
Male	2.78 ± 0.31	2.77 ± 0.21	2.19 ± 0.54	2.54 ± 0.39
Female	2.98 ± 0.42 *	2.79 ± 0.39	3.01 ± 0.44 *	2.65 ± 0.36 *
**Age**				
≤30	3.02 ± 0.23	2.86 ± 0.34	2.84 ± 0.40	2.91 ± 0.29
30–39	2.87 ± 0.36 *	2.78 ± 0.28 *	2.81 ± 0.35	2.88 ± 0.37
40–49	2.74 ± 0.19 *	2.80 ± 0.39 *	2.79 ± 0.26 *	2.81 ± 0.34
≥50	2.55 ± 0.27 *	2.61 ± 0.22 *	2.31 ± 0.37 *	2.74 ± 0.41 *
**HCWs subgroup**				
Physician	2.48 ± 0.51 *	2.54 ± 0.47 *	2.46 ± 0.40 *	2.39 ± 0.61 *
Graduate sanitary	3.01 ± 0.14	2.83 ± 0.22	2.76 ± 0.19	2.80 ± 0.30
Nurse and midwife	2.41 ± 0.21 *	2.39 ± 0.34 *	2.55 ± 0.47 *	2.40 ± 0.20 *
Healthcare assistance staff	2.74 ± 0.24	2.81 ± 0.20	2.77 ± 0.19	2.63 ± 0.31 *
Healthcare diagnostic staff	2.81 ± 0.33	2.79 ± 0.21	2.73 ± 0.31	2.78 ± 0.39
**Work environment**				
Surgery	2.31 ± 0.47 *	2.38 ± 0.56 *	2.40 ± 0.39 *	2.42 ± 0.50 *
Medicine	2.69 ± 0.33	2.74 ± 0.36	2.79 ± 0.29	2.71 ± 0.41
Services	2.49 ± 0.48 *	2.47 ± 0.35 *	2.51 ± 0.24 *	2.58 ± 0.39 *

* Statistically significant difference.

**Table 3:** Logistic regression.

Variables	DiscriminationOR (95% CI)	Acceptance of HIV/AIDSOR (95% CI)	Acceptance of Drug UsersOR (95% CI)	FearOR (95% CI)
**Gender**				
Female	1	1	1	1
Male	1.09 (1.06–1.12) *	1.03 (0.99–1.07)	1.02 (0.97–1.04)	1.05 (1.02–1.09) *
**Age**				
≤30	1	1	1	1
30–39	1.02 (0.96–1.05)	1.01 (0.97–1.05)	0.99 (0.95–1.04)	0.98(0.96–1.01)
40–49	1.03 (1.00–1.06) *	1.02 (0.97–1.06)	1.04 (1.00–1.09) *	1.04 (1.01–1.07) *
≥50	1.08 (1.05–1.12) *	1.06 (1.02–1.09) *	1.11 (1.06–1.17) *	1.07 (1.02–1.10) *
**Professional training**				
≤3 years	1	1	1	1
>4 years	1.05 (1.02–1.09) *	1.07 (1.01–1.13) *	1.04 (1.01–1.07) *	1.06 (1.02–1.10) *
**Years of services**				
≤10	1	1	1	1
11–25	1.04 (1.01–1.07) *	1.05 (1.02–1.08) *	1.03 (1.00–1.07) *	1.07 (1.03–1.11) *
≤26	1.03 (1.00–1.06) *	1.04 (1.01–1.09) *	1.01 (0.97–1.05)	1.06 (1.04–1.09) *

* Statistically significant difference.
